# Cognitive Biases of Consumers’ Risk Perception of Foodborne Diseases in China: Examining Anchoring Effect

**DOI:** 10.3390/ijerph16132268

**Published:** 2019-06-27

**Authors:** Lijie Shan, Shusai Wang, Linhai Wu, Fu-Sheng Tsai

**Affiliations:** 1 Institute for Food Safety Risk Management, Jiangnan University, Wuxi 214122, China; 2 School of Business, Jiangnan University, Wuxi 214122, China; 3 Department of Business Administration, Cheng Shiu University, Kaohsiung 83347, Taiwan; 4 Center for Environmental Toxin and Emerging-Contaminant Research, Cheng Shiu University, Kaohsiung 83347, Taiwan; 5 Super Micro Mass Research and Technology Center, Cheng Shiu University, Kaohsiung 83347, Taiwan

**Keywords:** foodborne disease, risk perception, anchoring effect, cognitive bias, intervention

## Abstract

Consumer cognitive biases arise from judgment and decision-making due to their limitations in information processing. As one of the important cognitive biases, the anchoring effect plays a significant role in interfering with consumers’ risk perception. With a stratified random approach, we collected survey data from 375 consumers in Wuxi, Jiangsu Province, China. Based on these data, this study attempted to analyze the anchoring effect in consumers’ risk perception of foodborne diseases (FBDs) and the differences in their perception before and after intervention in a contrast experiment using the anchoring index and the Wilcoxon signed-rank test. The results confirm the existence of the proposed anchoring effect. Moreover, the experimenter-provided anchor value, a history of FBD, and familiarity with FBD were found to be important factors influencing this anchoring effect. Therefore, improving consumers’ risk perception of FBD is critical to the long-term prevention of FBD risks by the government and consumers. The government should strengthen active monitoring, publicity, and education about FBD.

## 1. Introduction

In recent years, the frequency of foodborne disease (FBD) outbreaks has increased, which continues to be a major public health issue [1]. In 2013, the World Health Organization (WHO) published a first estimate of the global impact of foodborne illnesses, based on 2010 data. The data suggest that about 600 million cases of foodborne illnesses and 420,000 associated deaths occur globally each year, caused by 31 key known pathogens [2]. At present, there are more than 250 different foodborne diseases described [3], and more and more people are in the high-risk category of FBD [4]. FBD is very common in both developed and developing countries [5]. For instance, in England and Wales, there are 2,366,000 cases, 21,138 hospitalizations, and 718 deaths each year due to foodborne illness [6]. In China, an active surveillance of foodborne diseases in the first half of 2012 found that 1 in 6.5 persons had an FBD [7]. Moreover, the under-reporting rate of FBD may be as high as 95% in developing countries, including China [8]. At present, people have reached a consensus that FBD poses a serious danger for food safety.

The potential risk of FBD may occur at every step from farm to fork (e.g., food production, processing, transportation, and consumption). Consumers represent the endpoint of the food safety chain and, thus have been referred to as the “final line of defense” [9] in the prevention of FBD [10]. In reality, however, it has been reported that home is the place where the largest number of FBD cases occur in China [11]. One of the reasons for this is that many consumers are not aware of their vulnerability to FBD and underestimate their risk [12]. At present, Chinese consumers have started to pay more attention to FBDs and wish to obtain information on them [13]. However, consumer cognitive biases have resulted in their limited risk perception [14]. Consumers’ judgment of disease risk is not only affected by the objective facts of the disease itself, but more importantly by consumer psychology [15]. When consumers receive objective risk information about diseases, they will use an anchoring heuristic strategy [15]. Under uncertain circumstances, the judgment and decision result or target value are close to the initial information or initial value, namely the direction of the “anchor”, resulting in estimation bias. Only when the level of risk perceived by consumers reaches the objective risk level can they pay more attention to health-protective behavior and effectively reduce the risk of FBD [16,17]. Against this backdrop, it is meaningful to systematically investigate if there are anchoring effects during the process when consumers are sensing FBD risks. This could contribute by improving consumers’ knowledge about FBD risks, thus enabling them to care more about their health and to minimize cognitive biases related to FBD. Although there have been many scholarly works studying risk perception in food safety, less has been investigated regarding how cognitive bias could affect consumers’ decision making in issues related to food safety. Because risk perception is critical for consumption behavior and the anchoring effect is one of the most critical for risk perception, it is vital to have a deeper understanding of the anchoring effect’s influences on consumers’ food-safety-related perceptions and operations, such as those toward foodborne diseases. Therefore, we analyzed the perception of FBD risk and the differences in consumer perception before and after intervention in consumers in Wuxi, Jiangsu Province, China based on anchoring effect theory and Wilcoxon signed-rank test. Furthermore, the main factors affecting cognitive biases were examined to provide recommendations for the prevention of FBD.

## 2. Literature Review and Hypotheses

### 2.1. Literature Review

Most of the previous literature has focused on factors that influence consumers’ perception of FBD risk, such as individual characteristics, dietary behaviors, and knowledge of pathogens [18,19,20,21]. However, consumers have cognitive biases due to their own informational limitations. Unfortunately, there is very little literature dealing with the influence of cognitive bias in consumers’ perception of FBD risk. In one of the rare studies of this kind, Luber and Petra [22] suggested that consumers’ overconfidence led to cognitive biases in their risk perception of FBD. With the development of behavioral economics, more and more researchers have sought to explain the cognitive biases of consumers using the theory of behavioral economics. For example, Simon’s theory of bounded rationality suggests that consumers will largely deviate from rationality in judgment and decision-making due to uncertainty in the external environment and limited cognition, thus resulting in biases in judgment [23,24]. Based on prospect theory, Kahneman and Tversky [25] argued that consumers’ risk estimations tended to be intuitive and heuristic and were susceptible to the anchoring effect, thus leading to biases in judgment and decision-making.

The factors affecting consumer cognitive biases are complex. As one of the important cognitive biases [26], the anchoring effect was first proposed by Kahneman and Tversky [25], who pointed out that consumers did not always follow the rationality principle in making judgments and decisions, and often adjust their estimates and make final judgments based on subjective understanding and initial reference information (i.e., initial anchor). However, other factors such as an uncertain external environment and limited cognition make consumers unsure of the extent to which they can adjust their estimates, resulting in biased anchoring results. Scholars have called this the “anchoring effect” [27].

The initial studies of the anchoring effect were carried out as laboratory experiments on college students. Since students are a special group and the laboratory experimental environment is different from reality, the external validity of these studies was questioned. Subsequently, further studies verified the objective existence and relative stability of the anchoring effect in terms of economic decision-making [28], value evaluation [29], and bank lending [30]. In general, research on the anchoring effect is carried out in the following three aspects. First, the type of anchoring effect. For example, the anchoring effect elicited in the two-step research design by Kahneman and Tversky (1974) [25] is called the traditional anchoring effect (their study of the traditional anchoring effect involved instructing individuals to first make a relative judgment and then make an absolute judgment. For example, “Is the length of the Yangtze River greater or less than 6000 km?” and “What is the length of the Yangtze River?”). The anchoring effect induced by providing irrelevant information anchors separately in a one-step research design was called the basic anchoring effect by Wilson et al. (the one-step research design used by Wilson et al. (1996) in the study of the basic anchoring effect involved instructing individuals to make an absolute judgment directly—for example, “What is the length of the Yangtze River?”) [31]. According to the source of anchors, Epley and Gilovich [32] refer to the anchoring effect caused by externally provided anchor information as the experimenter-provided anchoring effect, and that induced by self-generated anchors as the self-generated anchoring effect. From the perspective of the source of anchors, the traditional and basic anchoring effects are both caused by externally provided anchors and can thus be classified as the experimenter-provided anchoring effect [33]. The second aspect of the anchoring effect that is researched is the selection of anchors. Most of the initial studies focused on investigating the anchoring effect using irrelevant anchors with fixed values. For example, Kahneman and Tversky (1974) used two irrelevant values as high and low anchors in a “wheel of fortune” experiment. In this experiment, subjects were instructed to spin the wheel and allow it to randomly stop at a number. Subjects were asked whether the percentage of African countries in the United Nations was greater or less than their number from the wheel of fortune. They were then asked to estimate the percentage of African countries. In fact, the “random” number was controlled to be “10” and “65” by the experimenter without the subject’s knowledge. Kahneman and Tversky found that the anchor values affected the final estimates and induced biases in judgment. Wilson et al. [31] used random ID numbers as anchors, demonstrating that final judgment was affected by anchor numbers presented alone, thus resulting in anchoring biases. In later studies, realistic problems were analyzed by using the relative relationship between variables or the certain variable changed with time series as anchors. For example, Huang [34] used the executive compensation of peer companies as the anchors and found that the executive compensation of peer companies had a positive predictive effect on the growth of executive compensation. The third aspect that is researched is the factors influencing the anchoring effect. Kahneman and Tversky suggested that anchor values were an important factor affecting the anchoring effect. However, Chapman and Johnson [35] believed that not all values had an anchoring effect, and that a significant anchoring effect would only occur when the respondent paid sufficient attention to the “anchor value” and the anchor value was compatible with the target value. Kaustia et al. [36] demonstrated that both respondents with or without professional knowledge were subject to anchoring effect, but the former was smaller. Zhang and Zhao [37] also reported that the familiarity of respondents with the risk was a factor that affected the anchoring effect. Specifically, the lower the familiarity, the easier it is for biases to occur in judgment, and the greater the anchoring effect. Guo et al. [38] found that the characteristics of respondents, such as age, gender, and cognitive needs, all had a significant impact on the anchoring effect during judgment.

In summary, many pioneering studies have been carried out on the anchoring effect, as one of the important cognitive biases. They have laid an important foundation for investigating the anchoring effect in consumers’ risk perception of FBD in the current study. However, the previous studies are limited to economic management, such as investment, evaluation, consumption, and management, and rarely address risk perception [36,39,40,41]. Moreover, the existing literature on how to reduce the anchoring effect is limited to experimental work. There is very little literature on interventions to deal with anchoring effects in the real world. However, further research is urgently required investigating whether there is an anchoring effect in the risk perception of consumers, which is a global challenge.

### 2.2. Hypotheses

At present, most Chinese consumers have not been educated about FBD. Their understanding of FBD is based on their own scientific literacy and information from the internet, journals, newspapers, and other media. According to the Tenth Chinese Scientific Literacy Survey conducted by the China Association of Science and Technology in 2018, the percentage of Chinese citizens with scientific literacy is only 8.47%, which considerably lags behind developed countries [42]. Due to the incomplete knowledge, consumers are bound to suffer cognitive biases to a certain degree, very likely including the anchoring effect. Although there are few reports on whether there is an anchoring effect in consumers’ risk perception of FBD, previous studies have confirmed that there is indeed an anchoring effect in the perception of a certain disease. For example, Bowen et al. [43] found that high experimenter-provided anchors caused high estimates in a study of breast cancer risk perception. Although the type of disease investigated is not the same as in the study of Bowen et al., the literature suggests that consumers generally make purchase decisions based on intuitive patterns, which are easily affected by the anchoring effect [15]. Therefore, it is hypothesized that:

**Hypothesis** **1.** *Consumers have an experimenter-provided anchoring effect in the risk perception of foodborne diseases.*

In a study of correlation between individual characteristics and risk-taking, Lauriola and Levin [44] found gender differences in the judgment of risk-taking behaviors because of the cognitive differences between males and females. Guo [45] suggested that men were generally more adventurous than women and were more likely to make irrational decisions. Guo et al. [38] observed that the anchoring effect in women was more significant than that in men when they studied peer-to-peer lending behavior. Therefore, it is hypothesized that:

**Hypothesis** **2.** *Gender is an important factor influencing the experimenter-provided anchoring effect in consumers’ risk perception of FBD.*

Individual experience is a vital influencing factor for decision-making, on which anchoring effect and adjustment constantly emerge to simplify the complex patterns of attitudes and thoughts for a reasonable estimation [46]. Parry et al. [47] observed differences in risk perception between people who had and had not experienced *Salmonella* food poisoning, where people who had experience of food poisoning perceived their personal risk from food poisoning to be higher than people who had no experience of food poisoning. Hence, risk experience affects risk perception and its potential biases. Therefore, it is hypothesized that:

**Hypothesis** **3.** *The experience of FBD is an important factor influencing the experimenter-provided anchoring effect in consumers’ risk perception of FBD.*

Brewer et al. [48] observed an anchoring effect on physicians’ judgment of patients’ prevalence of FBD, which was smaller than that on the patients’ self-judgment. Englich [49] reported that respondents that were highly familiar with the laws were almost unaffected by anchoring, and that the less familiar the respondents were with the laws, the more susceptible they were to anchors. If the respondent was very familiar with the object judgment, information related to the object could be directly extracted from their memory to make a correct judgment, and their estimates were hardly affected by the anchors. The anchoring effect on consumers’ risk perception of FBD may be affected by familiarity. The more familiar consumers are with the risk of FBD, the less susceptible they may be to the anchoring effect. Therefore, it is hypothesized that:

**Hypothesis** **4.** *Consumers’ familiarity with FBD is an important factor influencing the experimenter-provided anchoring effect in their risk perception of FBD.*

The experimenter-provided anchor has a certain impact on the traditional anchoring effect. The difference in the anchor value will directly affect the anchoring effect. A higher anchor value may cause a higher estimate, and vice versa [25]. Moreover, a relevant anchor produces a stronger anchoring effect than an irrelevant anchor [50]. Consumers’ estimates of the prevalence of FBD are one of the important measures of their risk perception of FBD [45]. Therefore, the prevalence of FBD can be used as an experimenter-provided anchor. Hence, it is hypothesized that:

**Hypothesis** **5.** *The experimenter-provided anchor value is an important factor influencing the experimenter-provided anchoring effect in consumers’ risk perception of FBD.*

In terms of reducing the negative effects of anchoring, the selective accessibility model proposed by Strack and Mussweiler [51] is used as an effective method to reduce the traditional anchoring effect. The higher the consistency in information between the anchor value and the object, the faster the respondent makes a judgment, and the greater the bias. Conversely, greater difference in information between the anchor and the object the judgment can reduce the anchoring biases to some extent [52]. Given the poor knowledge of consumers about FBD, consumers were instructed to read scientific information on FBD within a limited time as an intervention in this study. This was in an attempt to help consumers to think more about the accuracy and objectivity of external anchor information after gaining knowledge of FBD, so as to correct their misunderstanding of the risk of FBD, thus reducing the anchoring effect to a certain extent. Therefore, it is hypothesized that:

**Hypothesis** **6.** *Interventions can reduce the experimenter-provided anchoring effect in consumers’ risk perception of FBD.*

## 3. Materials and Methods

### 3.1. Experimental Design

#### 3.1.1. Selection of Anchor Values

According to Chapman and Johnson, an anchoring effect is commonly generated when the respondents pay sufficient attention to the anchors, and the anchoring effect will be more significant when the anchor value is compatible with the target estimate. For the “anchor” itself, there is no specific requirement on how it is generated or its value. The anchor value can be either rational or extreme, either informative or uninformative, and either (stratified) random or relevant [53]. Based on the approximate national FBD prevalence of 15% estimated by the Chinese National Center for Food Safety Risk Assessment [7], in this study 30% and 5% FBD prevalence were selected as high and low anchor values, respectively.

#### 3.1.2. Questionnaire Design

Based on the questionnaires proposed by Kahneman and Tversky [25], Nuccio et al. [54], and Jacowitz and Kahneman [55], three questionnaires were designed for the control, high anchor, and low anchor groups in this study, in order to verify the existence of self-generated and experimenter-provided anchoring effects. Furthermore, the questionnaires were revised based on a pre-survey conducted in Wuxi, Jiangsu Province, China to ensure validity. In the control group, surveyed consumers (hereinafter referred to as respondents) were given no related information and were asked to judge the prevalence of FBD directly. In the high and low anchor groups, respondents were informed of 30% and 5% FBD prevalence, respectively, and then instructed to make relative and absolute judgments. The pilot study was conducted on the January 2019 at Sanyang Plaza, Wuxi City, Jiangsu Province. A random sample of 30 respondents was selected. Among them, there were 13 males and 17 females. The results showed that 30 respondents had difficulty in judging the prevalence of foodborne diseases without any suggestion. Three respondents did not answer the question of the prevalence rate. Excluding the above three respondents, the estimated prevalence of 27 respondents was 3.082%, which was much smaller than the actual number. The pre-survey revealed that respondents in the control group found it difficult to give a specific value of FBD prevalence without any related information, often resulting in invalid questionnaires. Therefore, the final survey was carried out using the two sets of questionnaires of high and low anchor groups in order to investigate only the experimenter-provided anchoring effect.

Each questionnaire consisted of three parts. The first part was about respondents’ judgments on the concept, hazards, and prevalence of FBD. The second part provided respondents with knowledge about FBD (hereinafter referred to as the intervention), such as the concept, causes, and correct preventive measures of FBD, and mortality in the elderly and children. In the third part, respondents were asked to judge the risk of FBD and answer questions about the hazards, prevalence, and preventive measures of FBD after reading the information in the second part. The questions about hazards, prevalence, and preventive measures of FBD in the third part were the same as those in the first part. The aim was to uncover the differences in respondents’ risk perception of FBD before and after the intervention.

#### 3.1.3. Reliability

This study used SPSS 20.0 software (IBM, Armonk, NY, USA) to test the reliability of the risk perception scale. We selected the statements “foodborne diseases are major health risks” and “the elderly and children are more vulnerable to foodborne diseases” and “current measures to prevent foodborne diseases are scientific and safe” to assess the FBD perception levels of the consumers. The cognitive level was measured on a 7-point Likert scale. The higher the score, the stronger the consumer’s risk perception ability and the greater the perceived risk. Consumers made judgments on the prevalence of foodborne diseases by scoring the question “Do you think the prevalence of foodborne diseases is high?” on a 5-point Likert scale. (showed in Table 1)

### 3.2. Sampling and Survey Design

As one of China’s better-developed cities in the Yangtze river delta, Wuxi is home to a large number of migrants, including workers from relatively backward and remote areas. Local consumers are more aware of food safety than those from underdeveloped areas, and have a certain understanding of how to prevent food safety risks. The diversity of Wuxi consumer samples can more accurately distinguish consumers’ cognition of foodborne disease risk. Thus, to ensure the representativeness of the sample, the survey was conducted in five administrative districts of Wuxi (Liangxi, Binhu, Xishan, Huishan, and Xinwu Districts) by trained interviewers from the Institute for Food Safety Risk Management of Jiangnan University. Respondents were recruited by selecting every third consumer coming into view [56]. Specifically, the survey was carried out in shopping malls, large- and medium-sized supermarkets, markets of agricultural products, and food stores in different geographical areas in the above districts. The questionnaires were performed one-on-one on the spot. Eighty respondents were recruited in each district, amounting to a total of 400 respondents. In order to avoid systematic bias, respondents from each district were divided into two groups by drawing lots. As stratified random sampling ensures that each member in the group has an equal chance of being chosen, there is a considerable possibility that the sample had the same structure as the whole group. Note that in this study a representative region was selected without overt bias. However, we did not intend to guarantee true randomness. For example, we only recruited people who went to those markets, and not, for example, people who grow their own food, or people who did their shopping at times not attended by the surveyors.

The respondents were aged between 18 and 65. To further ensure the validity of the questionnaires, two questions defined by the two-step research design were used for screening in both questionnaires. For the purposes of this study, the two questions included “Do you think the prevalence of FBD is greater or less than 30%/5% in a year?” and “How many people out of 1000 do you think have FBD in a year?” If a respondent provided inconsistent answers for the two questions, then we treated the whole survey as invalid. In other words, a questionnaire was invalid when the prevalence of FBD was greater than 30% (5%) but another answer was less than 300 (50). Note that the wording of the survey asked the raters if they had FBD “because of eating foods,” thus excluding possibility that the raters misattributed chronic diarrhea or irritable bowel syndrome to FBD. Generally, the raters know the reason of illness in such cases. The formal survey was carried out between 15 and 30 January 2018. A total of 375 valid questionnaires were obtained, including 184 from the high anchor group and 191 from the low anchor group. A small gift of RMB 5 was provided to each respondent who completed the survey.

### 3.3. Methods

#### 3.3.1. Anchoring Index

Extensive and in-depth research has been conducted on how to measure the experimenter-provided anchoring effect. In most of these studies, data were transformed into logarithm values before the analysis of experimenter-provided anchoring effect. However, this conversion altered the original data, which led to distortion of the final results. In view of this, the experimenter-provided anchoring effect was measured in this study using the anchoring index (AI) proposed by Jacowitz and Kahneman [55], which could preserve the originality of the data and accurately detect the level of the experimenter-provided anchoring effect. The AI value reflects the degree of approach of the estimated value given by the respondents to the anchor value. The larger the AI, the greater the degree of approach, and the greater the anchoring effect. AI is calculated as

(1) $${\mathrm{AI}}_{high/low}=\frac{Median(high/low\;anchor\;group)-Median(control\;group)}{High/low\;anchor\;value-Median\;(control\;group)}.$$

As mentioned above, it was difficult for respondents to estimate the prevalence of FBD without any tips or information in the pre-survey, so estimates of the control group are not available. Due to the lack of data on the prevalence of FBD in Wuxi, the prevalence of FBD reported by the Chinese National Center for Food Safety Risk Assessment was used to replace the median estimate of the control group for the sake of simplicity. The modified equation is as follows:

(2) $${\mathrm{AI}}_{high/low}=\frac{Median(high/low\;anchor\;group)-Actual\;prevalence}{High/low\;anchor\;value-Actual\;prevalence}$$

The value of AI is generally between 0 and 1, but it may also be greater than 1. AI = 0 indicates that the estimate of the high/low anchor group is equal to the actual prevalence, representing no anchoring effect. AI = 1 indicates that the estimate of the high/low anchor group is equal to the anchor value of the other group, representing a significant anchoring effect. AI > 1 indicates that the estimate of the high/low anchor group is larger than the anchor value, representing an extremely significant experimenter-provided anchoring effect.

#### 3.3.2. Wilcoxon Signed-Rank Test

The differences in sample characteristics before and after intervention were tested mainly by the parametric *t*-test and the nonparametric Wilcoxon signed-rank test [57]. The advantage of the Wilcoxon signed-rank test over the *t*-test is that it does not require a normal distribution of the difference of paired data, but only a symmetrical distribution of the data. In this study, the existence of experimenter-provided anchoring effect in the risk perception of FBD was assessed by AI, and the influence of each factor on the experimenter-provided anchoring effect was verified by one-way analysis of variance. On this basis, the differences in respondents’ risk perception of FBD before and after intervention were assessed by the Wilcoxon signed-rank test.

## 4. Results and Discussion

### 4.1. Respondent Demographics

Demographics of the respondents in the high and low anchor groups are shown in Table 2. Overall, the statistics showed that sample fit a typical type of people living in a city like Wuxi. Among them, 49.60% of the respondents were male, which is basically consistent with the population distribution of Wuxi. Further, 53.73% of the respondents were aged between 18 and 40, 51.73% of the respondents had a bachelor’s degree or higher, and 57.33% were married. Among consumers, 6.93% were of low-income groups (annual household income of 50,000 yuan and less), 36.53% of respondents were of middle-low income groups (annual household income of 50,000–100,000 yuan), 19.47% of respondents were of middle-income groups (annual household income of 100,000–150,000 yuan), and 30.07% were of high-income groups (annual household income greater than 150,000 yuan). In addition, most of the respondents believed that they were in good health.

### 4.2. Existence of Anchoring Effect and Its Influencing Factors

The results based on AI and one-way analysis of variance are shown in Table 3 by SPSS 20.0. The statistical results of prevalence estimates given by respondents before and after the intervention are shown in Table 4.

The calculation results of Table 3 and Table 4 were further analyzed with the following findings:

(1) As can be seen from Table 3, the level of significance was less than 0.01 for the effect of the anchor value on the anchoring index in both the high and low anchor groups. In other words, the anchor value had a significant effect on the experimenter-provided anchoring effect. Also, as can be seen from Table 4, the mean prevalence estimates of the high and low anchor groups were 19.95% and 9.35%, respectively. Thus, a high anchor value induced a higher estimate, and vice versa. The anchoring index of the high anchor group was 0.330, which is significantly lower than the 0.565 of the low anchor group. The possible reason is that the reference to FBD prevalence given to respondents in the high anchor group went beyond what they believed about FBD, thereby reducing the credibility of the experimenter-provided anchor. This is consistent with the conclusions of Wilson et al. [31]. In addition, a high experimenter-provided anchor might be an implausible anchor and was less relevant to the prevalence of FBD, which reduced the anchoring effect. This is similar to findings of Oppenheimer et al. [50]. Therefore, an anchoring effect existed in respondents’ risk perception of FBD, and the value of the experimenter-provided anchor was an important factor affecting the experimenter-provided anchoring. Both H_1_ and H_5_ were confirmed.
(2) As shown in Table 3, the significance test of the influence of gender on the anchoring index was greater than 0.05 in both the high and low anchor groups, indicating that gender did not have a significant effect on the experimenter-provided anchoring effect. This is inconsistent with the results reported by Lauriola [44]. This may be attributable to non-significant differences in the scientific understanding of FBD between respondents of different genders as a result of asymmetric information due to the late start of the FBD surveillance system and low media attention paid to FBD in China. As a result, men and women obviously had similar sensitivity to risk perception of FBD. Therefore, gender was not a factor influencing the experimenter-provided anchoring effect in respondents’ risk perception of FBD. H_2_ was not confirmed.
(3) As can be seen from Table 3, the anchoring index increased from 0.423 to 0.627 with the decrease of familiarity with FBD in the low anchor group. This is consistent with the results of Blankenship et al. [58]. Conversely, the anchoring index decreased from 0.325 to 0.197 with the decrease of familiarity in the high anchor group. The possible reason is that the anchor value referenced in the high anchor group went beyond what respondents believed about FBD, so that they might have judged the prevalence without reference to the high anchor value. This is similar to the findings of Brewer [59]. As an implausible anchor, the high anchor used in this study enlarged the difference between the anchor value and information about the object of judgment acquired by the respondents, thus producing a contrast effect. This reduced the effect of the anchor value on respondents’ judgment of prevalence (i.e., reduced the anchoring effect). Therefore, the familiarity with FBD was an important factor influencing the experimenter-provided anchoring effect in respondents’ risk perception of FBD. H_4_ was confirmed.
(4) As shown in Table 3, the significance test of the influence of respondents’ experience with FBD on the anchoring index yielded values less than 0.05 in both the high and low anchor groups. This indicates that the experience of FBD had a significant effect on the experimenter-provided anchoring effect. The anchoring indexes of the respondents who had experience with FBD in the high and low anchor groups were 0.178 and 0.147, respectively, which were lower than those of other respondents. Moreover, the experimenter-provided anchoring effect under the low anchoring condition was greater than that under the high anchoring condition. Compared to other diseases studied, FBD has a lower health hazard and a higher prevalence. It is possible that respondents have different risk perceptions with regard to different diseases. Besides, respondents who have not experienced FBD might be more likely to underestimate the risk of FBD, resulting in a greater anchoring effect. Therefore, the experience of FBD was an important factor influencing the experimenter-provided anchoring effect in respondents’ risk perception of FBD. H_3_ was confirmed.

### 4.3. Differences in Risk Perception and the Experimenter-Provided Anchoring Effect before and after Intervention

The changes and difference test in respondents’ risk perception of FBD and the experimenter-provided anchoring effect before and after intervention as obtained by SPSS 20.0 are shown in Table 5 and Table 6, respectively. Both groups of respondents expressed concern about FBD before intervention. The concern was increased after intervention. However, there was no significant difference in their judgment of preventive measures before and after intervention. Specifically, the proportion of respondents who believed the prevalence of FBD was moderate or high increased from 69.11% to 85.87% in the low anchor group and from 67.93% to 81.52% in the high anchor group after intervention. Moreover, 19.90% of respondents in the low anchor group believed that the prevalence of FBD was lower than 5%, whereas nearly half of respondents in the high anchor group believed that the prevalence of FBD was higher than 30%. Thus, the experimenter-provided anchoring effect existed objectively in the risk perception of FBD and was not easy to eliminate. The proportion of respondents that considered FBD as a major health risk increased from 71.20% to 87.43% in the low anchor group and from 68.48% to 80.98% in the high anchor group after intervention. Moreover, 92.15% and 88.59% of the respondents in the low and high anchor groups, respectively, believed that the elderly and children were vulnerable to FBD after intervention, representing respective increases of 3.67% and 4.35%.

External anchoring effects were significantly different before and after the implementation of interventions. After the intervention, the estimate of the prevalence by consumers in the low anchoring effect group increased from 9.5% to 14.2%, and the external anchoring effect decreased from 0.565 to 0.08. The estimate of the prevalence among consumers in the high anchoring effect group rose from 19.95% to 24.75%, and external anchoring effects rose from 0.33 to 0.65. It can be seen that interventions that educate consumers about foodborne diseases can enhance consumer risk perception, but there is still a phenomenon of overestimating or underestimating prevalence. Meanwhile, the effect of the intervention on the external anchoring effect was also inconsistent. The external anchoring effect of the low anchor group was significantly reduced, while the high anchor group had the effect of increasing the external anchoring effect. It is possible that the knowledge of foodborne disease risk transmitted in a short period of time increased consumers’ worries about the risk of foodborne diseases, causing consumers to increase their estimated prevalence before the intervention given the anchor value. This also confirmed that Hypothesis 6 was not significantly supported. This suggests that knowledge of foodborne diseases cannot effectively reduce the external anchoring effect. The numerical anchor may have an important impact on the probability judgment of consumers’ foodborne disease risk, and is not easy to eliminate.

## 5. Conclusions

In this study, the cognitive biases and anchoring effect in consumers’ risk perception of FBD were investigated, and the effect of an intervention on the cognitive biases was assessed in a contrast experiment based on the anchoring effect theory. China has a large population as well as rapid economic and social development, but the overall integrity of the society is not high, although the Chinese government has made a series of great efforts to prevent foodborne disease outbreaks. In this context, the value of this study is its systematic investigation of the possible phenomenon of consumer anchoring effect in risk perception towards FBD, in order to build a theoretical and preliminary empirical reference for governmental and social organizations to adopt intervention practices.

The results confirmed the existence of an experimenter-provided anchoring effect in consumers’ risk perception of FBD. Moreover, this experimenter-provided anchoring effect was not affected by demographic differences, but by familiarity with FBD, FBD experience, and the anchor value. Consumers’ risk perception was improved to some extent with a short-term intervention, but there were still anchoring biases. This further confirms that the anchoring effect in the risk perception of FBD was stable and difficult to eliminate. Put differently, despite the effect of our intervention during the experiments, the participants as consumers in their own normal lives maintained the anchoring effect. Such results implied that even in real life when consumers are more active decision-makers, short-term interventions could not change consumers’ anchoring effect substantially—which proves the strong existence of the anchoring effect and the potential need for stronger and more long-term interventions (i.e., not the experimenter-provided ones, but those in real life). Therefore, improving consumers’ risk perception of FBD is critical to the long-term prevention of FBD risks by the government and consumers. The government should strengthen active monitoring, publicity, and education about FBD. Specifically, the information about FBD, including its actual prevalence and scientific meaning, should be disseminated to consumers through various media, such as the internet, television, and radio, to warn consumers of the objective risks of FBD. By publicizing the concept (i.e., to generate popular science knowledge) of foodborne disease prevalence and reports on measures to prevent foodborne diseases, consumers can effectively understand the risk of foodborne diseases, enhance the protection of their health, and reduce the occurrence of foodborne diseases. In addition, consumers should actively learn more scientific knowledge of FBD, in order to reduce judgment biases caused by the experimenter-provided anchoring effect, deal with the objective risks correctly and scientifically, and improve their risk perception.

However, there are certain limitations in this study. A major limitation is that not all variables that influence consumers’ cognitive biases regarding FBD (e.g., personality differences [60]) were considered due to the limited questionnaire length. These variables may also influence the anchoring effect in consumers’ risk perception of FBD. In addition, consumers were asked to read information about FBD within a limited time period as an intervention to influence their risk perception. However, it remains to be further investigated to what extent this intervention affects consumers’ risk perception of FBD.

## Figures and Tables

**Table 1:** Reliability statistics.

3 Indicators			2 Indicators		
Cronbach’s Alpha	Standardized Cronbach’s Alpha	Item Number	Cronbach’s Alpha	Standardized Cronbach’s Alpha	Item Number
0.387	0.433	3	0.763	0.765	2

**Table 2:** Demographic characteristic of participants.

Demographics	Categories	Low Anchor Group		High Anchor Group		Total Proportion (%)
		Sample Size	Percentage (%)	Sample Size	Percentage (%)	
Gender	Male	90	47.12	96	52.13	49.60
	Female	101	52.89	88	47.82	50.40
Age	18–28 years	58	30.37	91	46.91	39.73
	29–40 years	49	25.65	41	22.28	24.00
	41–55 years	77	40.31	43	23.37	32.00
	56–65 years	7	3.66	9	4.89	4.27
Marital status	Married	126	65.97	89	48.37	57.33
	Unmarried	65	34.03	95	51.63	42.67
Education	Lower than junior college	60	31.41	41	22.28	26.93
	Junior college	43	22.51	37	20.11	21.33
	Higher than junior college	88	46.07	106	57.61	51.73
Annual household income	50,000 yuan and less	9	4.71	17	9.24	6.93
	50,000–100,000 yuan	76	39.79	61	33.15	36.53
	100,000–150,000 yuan	39	20.42	34	18.48	19.47
	More than 150,000 yuan	67	35.08	72	39.13	30.07
Health status	Healthy	175	91.62	171	92.93	92.27
	Moderate	16	8.38	13	7.07	7.73
	Unhealthy	0	0	0	0	0

**Table 3:** Mean and standard deviation of anchoring index.

Anchoring Index	Description	Low Anchor Group		High Anchor Group	
		M	SD	M	SD
Gender	Male	0.50	1.13	0.27	1.25
	Female	0.63	1.16	0.40	1.21
Familiarity	High	0.39 **	0.60	0.38	1.16
	Moderate	0.53 **	1.15	0.32	1.36
	Low	0.93 **	1.19	0.28	0.99
Anchor value	—	0.57 ***	1.14	0.33 ***	1.22
History of FBD	Sometimes or often suffering from FBD due to diet	0.18 **	1.48	0.15 **	1.01
	Unknown or no history	0.64 **	1.05	1.06 **	1.68

Notes: ** indicates *p* < 0.05 for significance; *** indicates *p* < 0.01 for significance. FBD: foodborne disease.

**Table 4:** Estimates from high and low anchor groups (%).

Group	Pre-Intervention Estimates		Post-Intervention Estimates	
	Mean	Standard Deviation	Mean	Standard Deviation
Low anchor group (5%)	9.35	11.42	14.20	14.05
High anchor group (30%)	19.95	18.38	24.75	18.76

**Table 5:** Risk perception of respondents and anchoring index before and after intervention.

Statistical Characteristics	Category Index	Low Anchor Group		High Anchor Group	
		Pre-Intervention	Post-Intervention	Pre-Intervention	Post-Intervention
Prevalence of FBD	Very low	3.14%	2.62%	2.17	1.63
	Low	24.08%	6.81%	27.72	14.13
	Moderate	47.12%	37.70%	45.65	47.28
	High	21.99%	48.17%	22.28%	34.24%
	Very high	3.66%	4.71%	2.17%	2.72
FBD is a major health risk	Yes	71.20%	87.43%	68.48%	80.98%
	No	28.90%	12.57%	31.52%	19.02%
The elderly and children are vulnerable	Yes	88.48%	92.15%	84.24%	88.59%
	No	11.52%	7.85%	15.76%	11.41%
Is the prevalence greater than the anchor value?	Yes	64.92%	80.10%	33.70%	49.46%
	No	35.08%	19.90%	66.30%	50.54%
Anchoring index		0.57	0.08	0.33	0.65

**Table 6:** Wilcoxon signed-rank test.

Statistical Characteristics	Low Anchor Group		High Anchor Group	
	Z Statistic	Asymp. Sig. (2-Tailed)	Z Statistic	Asymp. Sig. (2-Tailed)
Prevalence of FBD	−6.95	0.00 ***	−4.23	0.00 ***
FBD is a major health risk	−7.66	0.00 ***	−5.18	0.00 ***
The elderly and children are vulnerable	−4.81	0.00 ***	−4.21	0.00 ***
Is the prevalence greater than the anchor value?	−4.64	0.00 ***	−4.53	0.00 ***
Prevalence of valuation	−7.72	0.00 ***	−5.72	0.00 ***
Anchoring index	−7.72	0.00 ***	−5.73	0.00 ***

Notes: *** indicates *p* < 0.01 for significance.
